# The acknowledgment of the treatment burden in the disease burden

**DOI:** 10.1093/rap/rkae005

**Published:** 2024-01-22

**Authors:** Diederik De Cock, Anne-Catherine Dens, David Walker, Sandra Robinson, Linn Karlsson

**Affiliations:** Biostatistics and Medical Informatics Research Group, Department of Public Health, Faculty of Medicine and Pharmacy, Vrije Universiteit Brussel (VUB), Brussels, Belgium; Department of Dermatology, University Hospitals KU Leuven, Leuven, Belgium; Research and Development, Northumbria Healthcare NHS Foundation Trust, North Shields, UK; Research and Development, Northumbria Healthcare NHS Foundation Trust, North Shields, UK; Pain and Rehabilitation Centre, Department of Medical and Health Sciences, Linköping University, Linköping, Sweden

**This editorial refers to ‘Exploring the treatment burden of disease-modifying anti-rheumatic drug monitoring in people with rheumatoid arthritis’, by Ryan *et al.*** https://doi.org/10.1093/rap/rkad054.

The qualitative study by Ryan *et al.* [1] shows the findings of interviews with 13 adults with RA on DMARDs and with three family members concerning perspectives of patients and family members on DMARD monitoring. Interviewees felt that drug monitoring was needed as part of DMARD treatment, but they also indicated the increased burden related to organizing and attending appointments for patients with RA and their family members.

In current RA recommendations, DMARDs are the cornerstone of RA treatment [2]. Rheumatologists who were part of the stakeholder group discussing the findings by Ryan *et al.* [1] expressed uncertainty in addressing the treatment burden caused by DMARD monitoring. They felt that in practice this was done reactively when patients missed visits for drug monitoring, for example. Such perspectives indicate that person-centred care remains a challenge in rheumatology care. Person-centred care underlines the importance of recognizing the patient as an expert on the illness and life situation. Including this patient experience is shown to be positively associated with clinical effectiveness and patient safety and can be an extra pillar of quality in health care [3]. In contrast, the adage ‘client is king’ is often reversed in health care, with patients struggling to find their voice in their treatment and follow-up plans. Although caregivers understand the positive impact of shared decision-making on the patient journey when managing chronic disease, its implementation is frequently missing in the routine consultation process. Shared decision-making remains a topic ill studied in rheumatology, and tools to support physicians and patients in this process are lacking [4]. In a survey on gout treatment, rheumatologists indicated that no treatment choice was offered to the patient, and if patients could make choices, they often had difficulty making them [5]. The lack of patient knowledge on topics related to treatment options was perceived as a main barrier.

The findings by Ryan *et al.* [1] prompt us to consider innovative solutions that can alleviate the treatment burden more proactively in RA management. Health-care innovations using nurse-led clinics, an integrated role of general practitioners and mobile health (mHealth) applications could be pivotal in improving the patient experience of daily clinical practice. Many believe that future rheumatology will be different from current standards [6]. Technical innovations, such at home monitoring of blood parameters, could also alleviate patient burden [7]. However, uptake of these solutions is hindered by many barriers [8, 9]. For example, patients in the study by Ryan *et al.* [1] indicated that face-to-face consultations remain necessary, and complete telemonitoring was regarded as neither adequate nor reliable by both the patient and the caregiver. Hybrid health-care delivery models that balance the advantages of technology with the need for personal contact should be explored. However, the pressure put on the lives of patients by the logistic pathway caused by the treatment itself remains understudied.

In conclusion, the study by Ryan *et al.* [1] highlights the treatment burden as a crucial aspect of the disease burden that health-care providers should factor into the shared decision-making process. It is vital to consider factors such as patient knowledge, language skills, health literacy, personal values and commitment to treatment adherence. Moreover, the study illuminates the positive aspect of drug monitoring, which can serve as an opportunity for a holistic assessment of well-being. Effective drugs alone are thus not enough; adherence to treatment and a patient-centric long-term management plan are essential for the health and well-being of patients suffering from chronic diseases. In the post-coronavirus disease 2019 landscape, patient expectations have evolved, emphasizing the need for greater shared decision-making, education and tools to tailor management plans to individual needs. As we continue to navigate the complexities of chronic disease management, the voices and experiences of patients must guide our efforts towards more effective, compassionate and patient-centred care.

## Data Availability

No new data were generated or analysed for this editorial.
